# Defining adolescent common mental disorders using electronic primary care data: a comparison with outcomes measured using the CIS-R

**DOI:** 10.1136/bmjopen-2016-013167

**Published:** 2016-12-01

**Authors:** Rosie P Cornish, Ann John, Andy Boyd, Kate Tilling, John Macleod

**Affiliations:** 1 School of Social and Community Medicine, University of Bristol, Bristol, UK; 2 Farr Institute, Swansea University Medical School, Swansea, UK; 3 Public Health Wales NHS Trust, Wales, UK; 4 Integrative Epidemiology Unit, University of Bristol, Bristol, UK

**Keywords:** depression, common mental disorders, ALSPAC, data linkage, electronic patient records, CIS-R

## Abstract

**Objective:**

To compare the prevalence of common mental disorders (CMDs) derived from data held in primary care records with that measured using the revised Clinical Interview Schedule (CIS-R) in order to assess the potential robustness of findings based only on routinely collected data.

**Design and setting:**

Comparison study using linkage between the Avon Longitudinal Study of Parents and Children (ALSPAC) and electronic primary care records.

**Participants:**

We studied 1562 adolescents who had completed the CIS-R in ALSPAC at age 17–18 years and had linkage established to their primary care records.

**Outcome measures:**

Outcome measures from ALSPAC were whether or not an individual met International Classification of Diseases-10 criteria for a diagnosis of (1) a CMD or, specifically, (2) depression. Lists of Read codes corresponding to diagnoses, symptoms and treatments were used to create 12 definitions of CMD and depression alone using the primary care data. We calculated sensitivities and specificities of these, using CIS-R definitions as the reference standard.

**Results:**

Sensitivities ranged from 5.2% to 24.3% for depression and from 3.8% to 19.2% for CMD. The specificities of all definitions were above 98% for depression and above 96% for CMD.

For both outcomes, the definition that included current diagnosis, treatment or symptoms identified the highest proportion of CIS-R cases.

**Conclusions:**

Most individuals meeting case definitions for CMD based on primary care data also met CIS-R case definitions. Conversely many individuals identified as cases using the CIS-R had no evidence of CMD in their clinical records. This suggests that clinical databases are likely to yield underestimates of the burden of CMD in the population. However, clinical records appear to yield valid diagnoses which may be useful for studying risk factors and consequences of CMD. The greatest epidemiological value may be obtained when information is available from survey and clinical records.

**Strengths and limitations of this study**

- We were able to successfully link data of Avon Longitudinal Study of Parents and Children (ALSPAC) individuals to their electronic primary care records.
- We were able to compare the relative performance of a number of different definitions of common mental disorders derived using routinely collected primary care data with measures derived from a standardised, and widely validated, survey assessment.
- Ours is the first study to investigate this issue among adolescents.
- Inclusion of symptom codes allowed us to examine the impact of excluding individuals not meeting diagnostic thresholds.
- Data for this study were only available for a subset of individuals who had completed the revised Clinical Interview Schedule in ALSPAC and who had consented to linkage to their routine health records.

## Background

Some evidence suggests a substantial increase in rates of depression and anxiety among children and adolescents in the UK in the past few decades,^1^ with recent figures estimating the prevalence to be 4% among 5–16-year olds^2^ and as high as 16% among those aged 16–24.^3^ These common mental disorders (CMDs) are among the top contributors to morbidity among adolescents^4^ and have many long-term consequences, impacting negatively on education, employment, quality of life and physical and mental health.^5^ Further, a large proportion of adults with these conditions first experience them during adolescence^4^ ^5^ and depression has been shown to be one of the leading causes of disability and premature death worldwide.^6^ Why rates of these disorders should have increased so substantially in young people and the most effective public health response is not clear. Reflecting this uncertainty, there have been calls for increased research into the true extent, causes, protective factors and effective treatments of these disorders among adolescents.^4^

In the UK and elsewhere, most people diagnosed with a CMD are either treated within primary care or referred from primary care to Child and Adolescent Mental Health Services (CAMHS), although a minority of adolescents may be referred directly through their school.^7^ ^8^ As such, electronic primary care data are a potentially valuable data source for carrying out research on these conditions. It is generally assumed that a proportion of individuals with disease in the population are undiagnosed for a number of reasons (the so called ‘clinical iceberg’ phenomenon). It has been argued that this undiagnosed—and thus untreated—proportion is likely to be substantial in the case of anxiety and depression.^7^ ^9^ For example, a large proportion of individuals with these conditions may not consult their general practitioner (GP) at all;^7^ further there is evidence that GPs may not always recognise these conditions^10^ ^11^ or may be unwilling to label individuals as having a disorder.^12^ Studies in the UK have also indicated that recording behaviour for depression and anxiety has changed over recent years, with GPs less likely to record diagnoses and more likely to record symptoms.^13–16^ For these reasons, prevalence and incidence estimates based on primary care data may be underestimates of the true burden of disease in the population.

In order to optimise the potential of using routinely collected primary care data in research, these issues need to be investigated and, where possible, quantified. A previous study demonstrated that patients with depression can be accurately identified for inclusion in a trial through their primary care records.^17^ More recently John *et al*^18^ investigated a number of algorithms for identifying adults with CMD using electronic primary care data using the five-item Mental Health Inventory (MHI-5) as a reference standard. The algorithms used different combinations of codes for current and historical diagnoses and symptoms, as well as treatment. They found that all case definitions had low sensitivity but high specificity.^18^ In the present study, we aimed to use electronic primary care records to evaluate a similar set of algorithms for identifying cases of CMD and depression alone, as ascertained by the revised Clinical Interview Schedule (CIS-R), among adolescents in the Avon Longitudinal Study of Parents and Children (ALSPAC).

## Methods

### Subjects

Subjects were those enrolled in ALSPAC who consented to linkage to their health records during the first stage of a recent consent campaign and whose GPs agreed to the extraction of their primary care record. ALSPAC is a prospective study of children born to just over 15 000 pregnant women living in and around Bristol, a city in the southwest of England, with due dates between 1 April 1991 and 31 December 1992.^19^ Detailed data were collected during the pregnancies and participants have been followed up since birth through questionnaires, clinics and linkage to routine data sets. (The ALSPAC website has a searchable data dictionary^20^ providing details of all available data.)

In ALSPAC, parental consent was mandatory until age 16. When the children reached legal adulthood (age 18), ALSPAC conducted a consent campaign to formally re-enrol the children into the study and, at the same time, to ask for consent for ALSPAC to link to their health and administrative records.^21^ The present study is based on a sample of 2806 children who had responded to this campaign providing consent to linkage to their health records by October 2012.

### Linkage between ALSPAC and the electronic primary care data

ALSPAC had previously established a linkage to participants' National Health Service (NHS) Patient Demographic Service record^19^ (which includes GP registration details). Using this linkage, we obtained GP registration details and derived a list of GP practices in which at least one consenting participant was currently registered. We then contacted the GPs seeking assent for the extraction of participants' records. An initial batch of assenting practices (those assenting before a specific date) was selected for a pilot extraction. From these, we identified practices that used a software system supplied by Egton Medical Information Systems (EMIS)^22^ or had installed practice record reporting software developed by Apollo Medical Systems (Apollo).^23^ ALSPAC commissioned EMIS and Apollo to extract the coded values of the participants' records (free-text components were not extracted) from these practices. The extracted records were anonymised and securely transferred into infrastructure developed at Swansea University as part of the Secure Anonymised Information Linkage (SAIL) databank.^24^ The extraction and transfer process—achieved using a trusted third-party approach and detailed below—used SAIL's ‘split file’ method and adhered to NHS standards of encryption and security. Once extracted, the GP software supplier split the data into separate files, one containing identifiers and one containing clinical information (coded information only; no free-text was extracted). They then assigned corresponding records within these files the same unique case ID number. The file of identifiers was encrypted and sent over the NHS N3 secure network to the NHS Wales Informatics Service (NWIS). NWIS converted the identifiers into an anonymised linkage field (ALF), an externally meaningless ID number, then sent a file containing ALF and case ID number into the SAIL infrastructure. The GP software suppliers then sent de-identified clinical data into the same infrastructure and these data were linked to the file containing ALF using the case ID number (which was subsequently dropped). Separately, the ALSPAC data linkage team created split files of ALSPAC data and sent these into SAIL infrastructure using the same mechanism. Within our secure working area, we were therefore able to link and analyse de-identified data.

### ALSPAC data

Depression and anxiety were measured using a self-administered, computerised version of the CIS-R^25^ completed during a study clinic attended when the participants were 17–18 years old. The CIS-R asks questions about a range of symptoms and can be used to assign International Classification of Diseases-10 (ICD-10) diagnoses of depression and anxiety disorders.^26^ ^27^ The outcomes used in this study were whether or not an individual met the criteria for a diagnosis of (1) depression, or (2) a CMD (depression, an anxiety disorder or both). Anxiety disorders in this case included generalised anxiety disorder, mixed anxiety and depression, panic disorders and phobic disorders. Although not a gold standard, we treated the CIS-R as the reference standard in this study because we wanted to be able to compare the relative performance of a number of different case definitions generated with the primary care data against a previously evaluated measure.

A number of sociodemographic factors known to be predictive of non-response were collected in ALSPAC during pregnancy and early infancy: the child's sex and ethnicity, maternal age and parity, and parental educational levels (classified as O level or lower, A level, and degree or higher).

### Electronic primary care data

The extracted primary care data consisted of Read codes V.2 (5 byte) together with associated dates. In an earlier study among adults, John *et al*^18^ identified sets of codes indicating diagnoses, symptoms and treatment (antidepressants, anxiolytics and hypnotics) for CMD. The latter study excluded phobic disorders; in the current study, we included these in our definition of anxiety disorders and therefore added relevant Read codes into our sets. We also added codes relating to disorders with onset specifically in childhood, a depression symptom code for ‘Loss of interest’ and codes for ‘O/E (observation of) panic attack’ and ‘C/O (complaining of) panic attack’. The codes we used are given in online supplementary table S1. The codes were used to create a number of definitions of depression and CMD. These definitions were similar to those investigated previously among adults:^18^ (1) Current treatment; (2) Current diagnosis (treated or untreated); (3) Current diagnosis, treated; (4) Current diagnosis or symptom (treated or untreated); (5) Current diagnosis or symptom, treated; (6) Current diagnosis or symptoms or treatment; (7) Historical or current diagnosis, currently treated; (8) Historical or current diagnosis or symptoms, currently treated; (9) Historical diagnosis, currently treated OR current diagnosis; (10) Historical diagnosis, currently treated OR current diagnosis or symptoms; (11) Historical or current diagnosis or symptoms, currently treated OR current diagnosis; and (12) Historical or current diagnosis or symptoms, currently treated OR current diagnosis or symptoms. Current was defined as being 6 months either side of the month in which the CIS-R was completed. The period after individuals had completed the CIS-R was included in order to take account of delays in consulting a GP or receiving a diagnosis and/or treatment. A historical diagnosis (or symptoms) was one that occurred at any time in an individual's GP record up to the period of interest. Although psychological therapies are the recommended first line of treatment for adolescents, these would generally be delivered through specialist mental health services and therefore not recorded in primary care data; thus, treatment in our definitions only referred to drugs. However, we did identify Read codes for referral to mental health services and used these to calculate referral rates.

10.1136/bmjopen-2016-013167.supp1 supplementary table Depression and CMD Read codes (version 2) used in case definitions

The GP data were also used to calculate the total number of GP consultations each individual had while aged 17–18 years. As in a previous study, consultation rates were calculated by excluding all Read codes relating to administration, hospitalisations and provision of services and multiple consultations within 1 day were counted as one.^28^

### Statistical analysis

We used Mann-Whitney U tests to compare consultation rates among individuals with and without CIS-R-defined CMD and depression. We compared each case definition of depression and CMD with the relevant ALSPAC-recorded outcome measured using the CIS-R. Sensitivity, specificity and predictive values were calculated using the CIS-R as the reference standard. Exact CIs were calculated based on binomial probabilities. All analyses were restricted to individuals who had a GP record up to at least 6 months after the month in which they completed the CIS-R. We also examined the records of individuals who were defined as having depression/CMD using the CIS-R but not using the GP data and vice versa; we confined this analysis to the case definition: current diagnosis or symptoms or treatment. Finally, t-tests were used to compare total CIS-R scores among those who met the most and least sensitive case definitions with those who did not (among those who were defined as having depression/CMD using the CIS-R). All analyses were carried out using Stata V.13.0.

## Results

There were 14 684 singletons and twins who enrolled in ALSPAC, who were alive at 1 year and had not subsequently withdrawn consent. Of these, 2806 had provided explicit consent for the extraction of their health records by October 2012 and were linked by the Health and Social Care Information Centre (HSCIC) to one of 523 GP practices. By August 2013, ALSPAC had gained the authorisation to extract records from 290 (55%) of these practices (16 (3%) had refused authorisation by this date and contact was ongoing with the remaining 217 (42%)). Among these 290 practices, 264 used either EMIS or Apollo software, or both. We extracted the records of 2249 participants from 181 practices (extracts from the remaining 83 practices could not be conducted due to technical/governance issues relating to the Apollo extract system or the underlying practice software system). Among these 2249 individuals, 1821 (83%) came to the study clinic at age 17–18 when the CIS-R was completed and 1657 completed it (74% of the original 2249). Of these, 1562 had a GP record up until at least 6 months after completing the CIS-R. Of these, 115 (7.4%) had a (CIS-R) diagnosis of depression and 213 (13.6%) had a CMD. The prevalence of CMD and depression were higher among female than male adolescents. Among female adolescents, 160/930 (17.2%) had CIS-R-defined CMD and 88 (9.5%) had depression. The figures for male adolescents were 53/632 (8.4%) for CMD and 27 (4.3%) for depression. Key characteristics of the ALSPAC-enrolled sample, those who completed the CIS-R and the individuals included in this study are given in table 1. Those who completed the CIS-R were more likely to be female, white and be the first-born child; their mother was more likely to be older and both parents more likely to have higher levels of education. These trends continued when restricting to those for whom we had linked GP data, although the differences were not as marked. Individuals included in the current study were slightly less likely to have CIS-R-defined depression or CMD than all ALSPAC individuals who completed the CIS-R.

**Table 1:** Comparison of the ALSPAC-enrolled sample, those who completed the CIS-R and the sample for whom we have linked GP data in terms of socioeconomic characteristics and CIS-R outcomes

	Singletons and twins enrolled in ALSPAC, alive at 1 year and who have not withdrawn from the study (n=14 684)*	Completed the CIS-R at 17–18 years (n=4563)*	Completed the CIS-R and in linked GP data set† (n=1562)*
Sex			
Male	7536 (51.3%)	1996 (43.7%)	632 (40.5%)
Female	7148 (48.7%)	2.567 (56.3%)	930 (59.5%)
Maternal age			
<20	650 (4.7%)	76 (1.8%)	12 (0.8%)
20–24	2688 (19.2%)	566 (13.2%)	159 (10.6%)
25–29	5403 (38.7%)	1621 (37.8%)	545 (36.3%)
30–34	3848 (27.5%)	1465 (34.2%)	567 (37.7%)
35+	1383 (9.9%)	561 (13.1%)	219 (14.6%)
Parity			
0	5769 (44.6%)	2011 (48.4%)	718 (48.7%)
1	4539 (35.1%)	1450 (34.9%)	526 (35.7%)
2	1849 (14.3%)	520 (12.5%)	179 (12.1%)
3+	767 (5.9%)	173 (4.2%)	51 (3.5%)
Ethnicity			
White	11 469 (95.0%)	3917 (95.7%)	1409 (96.1%)
Non-white	609 (5.0%)	176 (4.3%)	58 (4.0%)
Mother's education			
O level/lower	8021 (64.6%)	2195 (52.9%)	708 (47.8%)
A level	2792 (22.5%)	1162 (28.0%)	425 (28.7%)
Degree/higher	1599 (12.9%)	792 (19.1%)	347 (23.5%)
Father's education			
O level/lower	6662 (55.8%)	1875 (46.3%)	605 (41.5%)
A level	3105 (26.0%)	1167 (28.8%)	420 (28.8%)
Degree/higher	2168 (18.2%)	1009 (24.9%)	433 (29.7%)
CIS-R diagnosis of depression			
No	–	4203	1447
Yes	–	360 (7.9%)	115 (7.4%)
CIS-R diagnosis of CMD			
No	–	3884	1349
Yes	–	679 (14.9%)	213 (13.6%)

*The denominators vary because the variables come from different data sources and not all are complete.

†Up to at least 6 months beyond the date of completion of the CIS-R.

ALSPAC, Avon Longitudinal Study of Parents and Children; CIS-R, revised Clinical Interview Schedule; CMD, common mental disorder; GP, general practitioner.

Among the 1562 individuals included in this study, the median (IQR) number of GP consultations over the 2-year period (while aged 17–18) was 8 (4–15) among those who had a CMD and 6 (2–11) among those with no CMD as measured by the CIS-R (p<0.001), corresponding to—on average—one additional consultation per year for individuals with a CMD. The proportion of individuals who did not consult their GP at all during this 2-year period was correspondingly lower among those with a CMD (6.6%, compared with 9.7% among those with no CMD). Similarly, those with CIS-R-defined depression had higher consultation rates (median=10; IQR: 4–18) than those without depression (median=6; IQR: 3–11) (p<0.001).

### Comparison of the different case definitions

Table 2 shows sensitivities and specificities of the 12 cases definitions for depression based on GP records compared with depression as measured using the CIS-R. As expected, the sensitivities for all definitions were low, ranging from 5.2% (95% CI 1.9% to 11.0%) for current treated diagnosis to 24.3% (95% CI 16.8% to 33.2%) for current diagnosis or symptoms or treatment. The specificities were all above 98%, indicating that most of those meeting the case definitions were also identified as cases via the CIS-R. Adding historical diagnoses and/or symptoms had little or no impact on these results.

**Table 2:** Sensitivity and specificity of different definitions of depression (current defined as up to 6 months either side of date of completion of CIS-R)

		CIS-R diagnosis of depression		
Case definition		No	Yes	Total
Current treatment	No	1426 (98.6%)	100	1526
	Yes	21	15 (13.0%)	36
Current diagnosis	No	1441 (99.6%)	107	1548
	Yes	6	8 (7.0%)	14
Current diagnosis, treated	No	>99%*	109	*
	Yes	<5*	6 (5.2%)	*
Current diagnosis or symptoms	No	1426 (98.6%)	91	1517
	Yes	21	24 (20.9%)	45
Current diagnosis or symptoms, treated	No	1438 (99.4%)	104	1542
	Yes	9	11 (9.6%)	20
Current diagnosis or symptoms or treatment	No	1414 (97.7%)	87	1501
	Yes	33	28 (24.3%)	61
Historical or current diagnosis, currently treated	No	>99%*	107	*
	Yes	<5*	8 (7.0%)	*
Historical or current diagnosis or symptoms, currently treated	No	1438 (99.4%)	103	1541
	Yes	9	12 (10.4%)	21
Historical diagnosis, currently treated OR current diagnosis	No	1441 (99.6%)	105	1546
	Yes	6	10 (8.7%)	16
Historical diagnosis, currently treated OR current diagnosis or symptoms	No	1426 (98.6%)	90	1516
	Yes	21	25 (21.7%)	46
Historical or current diagnosis or symptoms, currently treated OR current diagnosis	No	1438 (99.4%)	102	1540
	Yes	9	13 (11.3%)	22
Historical or current diagnosis or symptoms, currently treated OR current diagnosis or symptoms	No	1426 (98.6%)	90	1516
	Yes	21	25 (21.7%)	46

*Numbers suppressed for disclosure control purposes.

CIS-R, revised Clinical Interview Schedule.

The results for CMD were similar to those for depression, with high specificities (all above 97%) and low sensitivities. These results are shown in table 3. Again, current treated diagnosis identified the lowest proportion of cases (3.8%; 95% CI 1.6% to 7.3%) and current diagnosis or symptoms or treatment the highest (19.2%; 95% CI 14.2% to 25.2%).

**Table 3:** Sensitivity and specificity of different definitions of CMD (current defined as up to 6 months either side of date of completion of CIS-R)

		CIS-R diagnosis of CMD (depression and/or anxiety disorder)		
Case definition		No	Yes	Total
Current treatment	No	1321 (97.9%)	189	1509
	Yes	29	24 (11.3%)	53
Current diagnosis	No	1332 (98.7%)	199	1531
	Yes	17	14 (6.6%)	31
Current diagnosis, treated	No	1340 (99.3%)	205	1545
	Yes	9	8 (3.8%)	17
Current diagnosis or symptoms	No	1318 (97.7%)	181	1499
	Yes	31	32 (15.0%)	63
Current diagnosis or symptoms, treated	No	1336 (99.0%)	198	1534
	Yes	13	15 (7.0%)	28
Current diagnosis or symptoms or treatment	No	1302 (96.5%)	172	1474
	Yes	47	41 (19.3%)	88
Historical or current diagnosis, currently treated	No	1339 (99.3%)	202	1541
	Yes	10	11 (5.2%)	21
Historical or current diagnosis or symptoms, currently treated	No	1334 (98.9%)	196	1530
	Yes	15	17 (8.0%)	32
Historical diagnosis, currently treated OR current diagnosis	No	1331 (98.7%)	196	1527
	Yes	18	17 (8.0%)	35
Historical diagnosis, currently treated OR current diagnosis or symptoms	No	1317 (97.6%)	179	1496
	Yes	32	34 (16.0%)	66
Historical or current diagnosis or symptoms, currently treated OR current diagnosis	No	1329 (98.5%)	192	1521
	Yes	20	21 (9.9%)	41
Historical or current diagnosis or symptoms, currently treated OR current diagnosis or symptoms	No	1316 (97.6%)	179	1495
	Yes	33	34 (16.0%)	67

CIS-R, revised Clinical Interview Schedule; CMD, common mental disorder.

There was evidence that sensitivities and specificities were higher for female than male adolescents, although the numbers were small and the CIs consequently wide, particularly for male adolescents. For example, for depression the sensitivity of current diagnosis or symptoms or treatment was 23.1% (16.8% to 30.4%) for female adolescents and 7.5% (2.1% to 18.2%) for male adolescents, and for CMD this definition had a sensitivity of 27.3% (18.3% to 37.8%) for female adolescents and 14.8% (4.2% to 33.7%) for male adolescents. For disclosure control reasons, the numbers cannot be shown.

The positive (PPVs) and negative predictive values (NPVs) for depression and CMD are shown in table 4. The NPVs were higher for depression alone (all above 93%) than for CMD (between 86% and 89%). For depression, case definitions including a diagnosis (current or historical) as well as current treatment gave the highest PPVs; the highest was 66.7% (95% CI 34.9% to 90.1%) for ‘current or historical diagnosis, currently treated’. The PPVs for CMD were generally lower and ranged from 45.2% for current treatment to 53.6% for current diagnosis or symptoms, treated.

**Table 4:** Positive (PPV) and negative predictive values (NPV) for different case definitions of depression and CMD

	Depression		Common mental disorder	
Case definition	NPV (95% CI)	PPV (95% CI)	NPV (95% CI)	PPV (95% CI)
Current treatment	93.4% (92.1% to 94.6%)	41.7% (25.5% to 59.2%)	87.5% (85.1% to 89.1%)	45.3% (31.6% to 60.0%)
Current diagnosis	93.1% (91.7% to 94.3%)	57.1% (28.9% to 82.3%)	87.0% (85.2% to 88.6%)	45.2% (27.3% to 64.0%)
Current diagnosis, treated	93.0% (91.6% to 94.2%)	60.0% (26.2% to 87.8%)	86.7% (84.9% to 88.4%)	47.1% (23.0% to 72.2%)
Current diagnosis or symptoms	94.0% (92.7% to 95.1%)	53.3% (37.9% to 69.3%)	87.9% (86.2% to 89.5%)	50.8% (37.9% to 63.6%)
Current diagnosis or symptoms, treated	93.3% (91.9% to 94.5%)	45.0% (23.1% to 68.5%)	87.1% (85.3% to 88.7%)	53.6% (33.9% to 72.5%)
Current diagnosis or symptoms or treatment	94.2% (92.9% to 95.3%)	45.9% (33.1% to 59.2%)	88.3% (86.6% to 89.9%)	46.6% (35.9% to 57.5%)
Historical or current diagnosis, currently treated	93.1% (91.7% to 94.3%)	66.7% (34.9% to 90.1%)	86.9% (85.1% to 88.5%)	52.4% (29.8% to 74.3%)
Historical or current diagnosis or symptoms, currently treated	93.3% (92.0% to 94.5%)	57.1% (34.0% to 78.2%)	87.2% (85.4% to 88.8%)	53.1% (34.7% to 70.9%)
Historical diagnosis, currently treated OR current diagnosis	93.2% (91.8% to 94.4%)	62.5% (35.4% to 84.8%)	87.2% (85.4% to 88.8%)	48.6% (31.4% to 66.0%)
Historical diagnosis, currently treated OR current diagnosis or symptoms	94.1% (92.8% to 95.2%)	54.3% (39.0% to 69.1%)	88.0% (86.3% to 89.6%)	51.5% (38.9% to 64.0%)
Historical or current diagnosis or symptoms, currently treated OR current diagnosis	93.4% (92.0% to 94.6%)	59.1% (36.4% to 79.2%)	87.4% (85.6% to 89.0%)	51.2% (35.1% to 67.1%)
Historical or current diagnosis or symptoms, currently treated OR current diagnosis or symptoms	94.1% (92.8% to 95.2%)	54.3% (39.0% to 69.1%)	88.0% (86.3% to 89.6%)	50.7% (38.2% to 63.2%)

### Referrals to mental health services

Among the 61 individuals with a record of a diagnosis, symptoms or treatment for depression, 10 (16%) had a current referral to mental health services; similarly, among the 88 with a GP record for a diagnosis, symptoms or treatment for a CMD, 11 (13%) had a current referral.

### ‘CIS-R negative’ individuals identified as cases in their GP record (‘false positives’)

#### Depression

There were 33 individuals who had a current diagnosis, symptoms or treatment for depression but were not identified as having depression using the CIS-R. Possible explanations were found for over 75% of these discrepancies (total number suppressed for disclosure control purposes). Specifically, five (15.2%) were either identified as having an anxiety disorder (but not depression) on the CIS-R or were receiving an antidepressant but had an anxiety diagnosis in their GP record. A further nine (27.3%) were receiving treatment when they completed the CIS-R and, as such, may have been asymptomatic at the time (assuming the treatment was effective). An additional nine (27.3%) had their first record of depression after they completed the CIS-R and thus may have developed their illness after completing the survey. Finally, a small number of individuals were receiving amitriptyline for pain relief (small numbers suppressed).

#### Common mental disorders

Similarly, potential explanations were found for over 60% (number suppressed) of these discrepancies: 11 (23.4%) of the 47 individuals who had a current diagnosis, symptoms or treatment for CMD but were not identified as such with the CIS-R were receiving treatment when they completed the CIS-R; 15 (31.9%) had their first diagnosis, symptom or treatment recorded after completing the CIS-R; and a small number were either receiving hydroxyzine as an antihistamine or amitriptyline for pain relief (small numbers suppressed).

### CIS-R cases who did not meet a case definition based on their GP records (‘false negatives’)

#### Depression

There were 87 individuals (64 females, 23 males) who had no current diagnosis, symptoms or treatment but who had CIS-R-defined depression. Among these, 40 (46%) had a relevant entry at some point in their GP record (this accounted for 48% of the female adolescents who were ‘false negatives’ and 39% of the male adolescents). Specifically, nine (10.3%) had one or more of a historical diagnosis, symptom or treatment; eight (9.2%) had either a diagnosis, symptom or treatment for anxiety but not depression or had a record of a referral to mental health services, and 16 (18.4%) had a record of either a diagnosis, symptoms or treatment more than 6 months after completion of the CIS-R (11 for depression, five for anxiety). An additional seven (8.0%) had a record of either ‘Tiredness symptom’, ‘Tired all the time’ (symptom or diagnosis) or ‘Fatigue’, although in most cases this was greater than a year either before or after they completed this CIS-R (small numbers suppressed).

#### Common mental disorders

The patterns were similar for CMD. Among the 172 individuals (123 females, 49 males) who had no current diagnosis, symptoms or treatment but had a CIS-R-defined CMD, 70 (41%) had a relevant entry in their GP record: 18 (10.5%) had a historical diagnosis, symptoms, or treatment; 37 (21.5%) had a record of a diagnosis, symptoms or treatment more than 6 months after completion of the CIS-R or had a record of a referral to mental health services; and 15 (8.7%) had a record of tiredness/fatigue. As was the case for depression, these explanations accounted for a larger proportion of the female than male ‘false negatives’ (44% compared with 33%).

### CIS-R scores

Among individuals identified as having depression or CMD using the CIS-R, total CIS-R scores were higher, on average, among those identified as cases using the GP data than those who were not. Results for two of the case definitions are shown in table 5.

**Table 5:** Mean (SD) total CIS-R scores among individuals meeting the CIS-R criteria for a diagnosis of depression or CMD according to whether or not they also met case definitions based on GP data

		Met case definition		
Outcome	Case definition	Yes	No	Difference in means (95% CI)
CMD	Current diagnosis, treated	26 (8.4)	16 (7.0)	−10 (−15 to −5); p<0.001
	Current diagnosis, symptoms or treatment	21 (8.1)	15 (6.6)	−5 (−8 to −3); p<0.001
Depression	Current diagnosis, treated	28 (4.9)	19 (6.4)	−9 (−14 to −4); p=0.001
	Current diagnosis, symptoms or treatment	23 (6.7)	19 (6.3)	−4 (−7 to −2); p=0.002

CIS-R, revised Clinical Interview Schedule; CMD, common mental disorder; GP, general practitioner.

## Discussion

In this study, we compared case definitions for CMD based on information contained in linked primary care data with case definitions derived from the CIS-R among adolescents aged 17–18 years. We have demonstrated that, taking the latter as the reference standard, definitions based on primary care data have high specificity but low sensitivity. This would lead to substantially lower estimates of prevalence in clinical compared with survey data. Definitions consisting of a treated diagnosis had the highest specificities but very low sensitivities; this is unsurprising, as these are likely to identify the more severe cases. This is supported by our results, as individuals meeting this case definition had higher CIS-R scores (on average) than those meeting the most sensitive definition.

### Strengths and limitations

As we have only linked to GP records from a subset of GP practices, there are some limitations in terms of the data. In particular, the completeness of the extracted records was dependent on the length of time a particular individual had been registered at a GP practice (or GP practices) using EMIS or Apollo software and, among those moving to a relevant practice at some point during childhood, what level of detail from their historical record was transferred to this new practice. Although historical records may be complete for some individuals, this will not be the case for everyone. As such, estimates of the sensitivity and PPV for definitions including historical diagnoses or symptoms may be underestimates. In addition, the GP data we extracted contained only coded information (Read codes and, where applicable, associated values); free-text information was not extracted. This could potentially lead to missed cases.^29^ However, this will be the case for most studies using routine GP data, as free-text information is not generally available for research use.^29^

Individuals included in this study were those who attended the ALSPAC clinic when the CIS-R was administered and were early responders to a consent request for linkage to health records. It is known that those who continue to participate in ALSPAC are more likely to be female, white individuals, and less likely to live in low-income households.^19^ The prevalence of CMD is therefore likely to be different among those who were not included in this study. Indeed, the prevalence of CMD as measured using the CIS-R is around 3% lower in our study—among female and male adolescents—than that estimated among 16–24-year olds in the 2007 adult psychiatric survey in England^3^ (carried out at around the same time as the CIS-R was administered in ALSPAC). This may reflect the narrower age range as well as the fact that our sample under-represents the lower socioeconomic groups. Further, it is possible that individuals who did not take part have different behaviours in terms of GP consultation rates and general help-seeking behaviour; this would affect the sensitivity and specificity of the different case definitions. However, the relative performance of the different case definitions in terms of their specificity and sensitivity is unlikely to have been affected.

The CIS-R is a standardised assessment used to measure CMDs. It has been shown to be reliable, either when administered by a trained interviewer or self-completed using the computerised version,^25^ ^30^ and is widely used, including in the National Survey of Adult Psychiatric Morbidity in England.^3^ Having said this, some studies have suggested that the CIS-R does not perform well in terms of deriving valid diagnoses^27^ and that tools like the CIS-R are not measuring the same thing as a clinical assessment.^31^ Indeed, the measurement of mental health outcomes in surveys is seen as a particular challenge in psychiatric epidemiology.^26^ ^32^ In the present study, we used the CIS-R as a comparator to allow us to illustrate apparent differences in prevalence between our two data sources and across the different case definitions, using widely understood diagnostic measures such as sensitivity and specificity. We are not arguing in this context for the greater validity of one data source over another; rather we are illustrating that these are different and discussing the reasons for and implications of this. For a number of reasons, clinical databases are likely to produce underestimates of prevalence. Prevalence estimates derived from surveys, such as those derived from ALSPAC may also be subject to bias due to selective participation. In studies such as ALSPAC where survey measures and linked data are available—at least for a subset of individuals—bias could be reduced by combining the two sources of information in some way.

### Comparison with other work

Our study confirms previous findings indicating that only a relatively small proportion of individuals with a CMD will receive a diagnosis or treatment, perhaps because they do not report their symptoms to a GP, or because their GP is either unwilling to label adolescents as having these conditions^12^ or fails to recognise them,^7^ ^9–11^ despite the fact that, on average, these individuals visit their GP more often than those without mental health problems (CMD).^33^ This, as highlighted in a recent review,^34^ has clear implications in terms of the need for improvements in GP training as well as closer working and access to specialist services.^34^ In a recent study, John *et al*^18^ validated primary care-based definitions of adult CMD using the MHI-5, a five-item subscale of the 36-item Short-Form Health Survey Questionnaire (SF-36) shown to be useful as a screening tool for CMD.^35^ ^36^ As here, they^18^ found that their definitions had high specificities but low sensitivities. However, in contrast to our study in adolescents, John *et al*^18^ found that current treatment had a relatively high sensitivity. This is not surprising as the recommended first line of treatment for depression in young people is psychological therapy^37^ (although in 2015 the guidelines were updated to suggest considering the use of combined therapy for moderate to severe depression^37^) and anxiolytics are only recommended for use in children for acute anxiety and associated insomnia;^38^ further, the British National Formulary for Children states that the use of hypnotics is rarely justified and should only be used as a one-off for sedation.^38^ Conversely, among adults with moderate-to-severe depression, a combination of psychological therapy plus medication is recommended^39^ and drug treatments are recommended for adults with anxiety if they have not responded to psychological therapies.^40^ Unfortunately, although GP referrals to specialist mental health services are recorded in GP data, we did not have information on treatment received (if any) within these or similar services, either via a GP referral or accessed via other means. This is a further limitation of using primary care data to identify adolescents with CMD.

## Conclusions

In summary, we have found that primary care-based definitions that use a combination of diagnoses, symptoms and treatment provide the most sensitive definitions for identifying adolescents with depression and CMD in population-based studies. These definitions have high specificity and would therefore be useful in studies using primary care data to identify risk factors for these conditions.^41^ The estimates of sensitivity and specificity could also be used to adjust estimates of incidence or prevalence using GP databases. Further research is needed to find ways to minimise bias in studies where survey and linked primary care data are available.
