# Supplementary material for: Defining adolescent common mental disorders using electronic primary care data: a comparison with outcomes measured using the CIS-R
Source: BMJ Open. 2016 Dec 1;6(12):e013167. doi: 10.1136/bmjopen-2016-013167 (PMC5168670; doi:10.1136/bmjopen-2016-013167)
Supplement: supplementary table — Depression and CMD Read codes (version 2) used in case definitions [file bmjopen-2016-013167supp_table.pdf]

Supplementary Table 1: Depression and CMD Read codes (version 2) used in case definitions

Depression diagnoses

Eu32.	[X]Depressive episode
Eu320	[X]Mild depressive episode
Eu321	[X]Moderate depressive episode
Eu322	[X]Severe depressive episode without psychotic symptoms
Eu324	[X]Mild depression
Eu32y	[X]Other depressive episodes
Eu32z	[X]Depressive episode, unspecified
Eu33.	[X]Recurrent depressive disorder
Eu330	[X]Recurrent depressive disorder, current episode mild
Eu331	[X]Recurrent depressive disorder, current episode moderate
Eu332	[X]Recurrent depressive disorder, current episode severe without psychotic symptoms
Eu334	[X]Recurrent depressive disorder, currently in remission
Eu33y	[X]Other recurrent depressive disorders
Eu33z	[X]Recurrent depressive disorder, unspecified
Eu341	[X]Dysthymia
E118.	Seasonal affective disorder
E135.	Agitated depression
E2B..	Depressive disorder NEC
E2B1.	Chronic depression
E291.	Prolonged depressive reaction
E204.	Neurotic depression reactive type
E2B0.	Postviral depression
E112.	Single major depressive episode
E1120	Single major depressive episode, unspecified
E1121	Single major depressive episode, mild
E1122	Single major depressive episode, moderate
E1123	Single major depressive episode, severe, without psychosis
E1125	Single major depressive episode, partial or unspecied remission
E1126	Single major depressive episode, in full remission
E112z	Single major depressive episode NOS
E113.	Recurrent major depressive episode
E1130	Recurrent major depressive episodes, unspecified
E1131	Recurrent major depressive episodes, mild
E1132	Recurrent major depressive episodes, moderate
E1133	Recurrent major depressive episodes, severe, no psychosis
E1135	Recurrent major depressive episodes, partial/unspecified remission
E1136	Recurrent major depressive episodes, in full remission
E1137	Recurrent depression
E113z	Recurrent major depressive episode NOS

Anxiety diagnoses

Eu41.	[X]Other anxiety disorders
Eu410	[X]Panic disorder [episodic paroxysmal anxiety]
Eu411	[X]Generalized anxiety disorder
Eu413	[X]Other mixed anxiety disorders

Eu41y [X]Other specified anxiety disorders
Eu41z [X]Anxiety disorder, unspecified
E200. Anxiety states
E2000 Anxiety state unspecified
E2001 Panic disorder
E2002 Generalised anxiety disorder
E2004 Chronic anxiety
E2005 Recurrent anxiety
E200z Anxiety state NOS
E202. Phobic disorders
Eu40. Phobic anxiety disorder
Eu930 Separation anxiety disorder of childhood
Eu931 Phobic anxiety disorder of childhood
Eu932 Social anxiety disorder of childhood

Mixed depression and anxiety diagnoses
E2003 Anxiety with depression
Eu412 [X]Mixed anxiety and depressive disorder

Depression symptoms
1B17. Depressed
1B1U. Symptoms of depression
1BQ.. Loss of capacity for enjoyment
1BT.. Depressed mood
1BU.. Loss of hope for the future
2257. O/E – depressed
1BP.. Loss of interest

Anxiety symptoms
1B13. Anxiousness
2258. O/E - anxious
1B12. Nerves, nervousness
R2y2. (D) nervousness
2259. O/E nervous
225J. O/E panic attack
1B1V. C/O panic attack

Drugs

Antidepressants
d71.. Amitriptyline hydrochloride
d72.. Butriptyline - discontinued
d73.. Clomipramine hydrochloride
d74.. Desipramine hydrochloride
d75.. Dosulepin Hydrochloride
d76.. Doxepin
d77.. Imipramine hydrochloride
d78.. Iprindole
d79.. Lofepramine

d7a.. Maprotiline hydrochloride
d7b.. Mianserin hydrochloride
d7c.. Nortriptyline
d7d.. Protriptyline hydrochloride
d7e.. Trazadone hydrochloride
d7f.. Trimipramine
d7g.. Viloxazine hydrochloride
d7h.. Amoxapine
d81.. Phenelzine
d83.. Isocarboxazid
d84.. Tranylcypromine
d85.. Moclobemide
d91.. Compound Antidepressants A-Z
da1.. Flupentixol [Antidepressant]
da2.. Tryptophan
da3.. Fluvoxamine Maleate
da4.. Fluoxetine hydrochloride
da5.. Sertraline hydrochloride
da6.. Paroxetine hydrochloride
da7.. Venlafaxine
da9.. Citalopram
daA.. Reboxetine
daB.. Mirtazapine
daC.. Escitalopram
daD.. Agomelatine
gde.. Duloxetine

Hypnotics
d11.. Chloral hydrate
d12.. Clomethiazole edisylate (hypnotic)
d13.. Dichloralphenazone - discontinued
d14.. Flumtrazepam - discontinued
d15.. Flurazepam
d16.. Loprazolam
d17.. Lormetazepam
d18.. Nitrazepam
d1a.. Temazepam (hynotic)
d1b.. Triazolam - discontinued
d1c.. Triclofos sodium
d1d.. Zopiclone
d1f.. Zolpidem
d1g.. Zaleplon
d1h.. Melatonin
d1i.. Dexmedetomidine

Anxiolytics
d21.. Diazepam
d22.. Alprazolam

d23.. Bromazepam
d24.. Chlordiazepoxide
d25.. Chlormezanone
d26.. Clobazam
d27.. Clorazepate dipotassium
d28.. Hydroxyzine hcl (anxiolytic)
d29.. Ketazolam - discontinued
d2a.. Lorazepam (anxiolytic)
d2b.. Medazepam - discontinued
d2c.. Meprobamate
d2d.. Oxazepam
d2f.. Buspirone hydrocholoride
d2g.. Flumazenil
